# Radiographic and Tumor Biomarker Response to Radiotherapy for Recurrent Granulosa Cell Tumor of the Ovary

**DOI:** 10.7759/cureus.13154

**Published:** 2021-02-05

**Authors:** Sarah A Singh, Basem Dahshan, Rebecca F Krc, David M McDermott, Geraldine M Jacobson

**Affiliations:** 1 Radiation Oncology, West Virginia University School of Medicine, Morgantown, USA

**Keywords:** granulosa cell tumor, palliative radiation therapy, recurrent granulosa cell tumor ovary, ovarian cancer, inhibin b, recurrent ovarian cancer

## Abstract

Granulosa cell tumors (GCTs) of the ovary are rare, comprising less than 5% of all malignant ovarian neoplasms. While generally considered indolent, GCTs have a tendency for metastasis and delayed relapse, with recurrence developing in 20%-50%. Recurrent or metastatic disease is associated with aggressive behavior and a poor prognosis, as nearly 70% of patients developing recurrence will eventually succumb to their disease. The optimal management of relapsed disease is controversial. Initial salvage therapy typically involves surgical debulking followed by cisplatin-based chemotherapy. Unfortunately, tumor responses are durable for less than half of patients treated with this regimen. Radiation therapy is an attractive option for providing rapid palliation and improving local control without the morbidity of additional surgery or chemotherapy. Here we describe a case of multiply recurrent, rapidly growing intraperitoneal GCT refractory to repeated surgical debulking and several lines of systemic therapy. The patient was treated with two courses of palliative radiotherapy and achieved rapid symptomatic relief, achieving over a 90% reduction in tumor volume. Serum concentration of inhibin B, often inappropriately elevated in patients with GCT, decreased by 98% following irradiation with no interim systemic therapy. At one-year follow-up, the patient has no evidence of radiographic or biochemical recurrence.

## Introduction

Malignant sex cord-stromal tumors (MSCST) of the ovary are rare, comprising less than 8% of all malignant ovarian neoplasms. The most common MSCST subtype is granulosa cell tumor (GCT), accounting for over 90% of MSCSTs and 2%-5% of all malignant ovarian neoplasms [1]. There are two subtypes of GCT, adult and juvenile, which vary in clinical presentation, histology, and behavior. The adult subtype of GCT accounts for 95% of cases, occurring at a median age of 50. The juvenile subtype, accounting for 5% of all cases, occurs in prepubescent females. Histopathologically, GCTs are characterized by “coffee-bean” nuclei and Call-Exner bodies [2]. Somatic mutations in the forkhead box protein L2 (FOXL2) gene, which plays a role in granulosa cell differentiation, are present in over 97% of adult GCTs. In contrast, this mutation is observed in only 10% of juvenile GCTs, 21% of thecomas, and is generally absent in other malignant ovarian tumor subtypes [3].

Granulosa cells produce the hormone inhibin, an estradiol-stimulating growth factor that negatively regulates follicle-stimulating hormone (FSH). Serum concentrations of inhibin B, often inappropriately elevated in patients with GCT, reflect the extent of tumor burden and serve as a useful biomarker for treatment response and disease recurrence [4]. GCT masses can be solid or cystic and have capacity for immense growth, measuring up to 40cm. As such, patients commonly present with symptoms of mass effect, including abdominal pain, distension, and early satiety [5]. The majority of patients present with early-stage disease and have an excellent prognosis, with five-year survival rates exceeding 90%. In contrast, the five-year overall survival rate for patients with advanced stage or recurrent disease is less than 33% [6].

Surgery is the mainstay of initial therapy and tumor staging. The current standard of care is total abdominal hysterectomy with bilateral salpingo-oophorectomy and peritoneal biopsy, although more conservative surgeries may be considered for women of child-bearing age interested in fertility preservation. Surgery alone is considered adequate for small early-stage tumors with a five-year disease-free survival exceeding 90%, although some experts advocate for the use of adjuvant chemotherapy when high-risk features are present [7]. Risk factors for recurrence after surgery include older age, large tumor size, advanced stage, tumor rupture, lymphovascular space invasion (LVSI), nuclear atypia, and high mitotic index [5]. While GCTs are generally considered indolent tumors, they have a tendency for metastasis and delayed relapse, with recurrence developing in 20%-50% of patients depending on initial stage [6]. Relapse most commonly occurs in the peritoneal cavity at a median time of 4-6 years from initial diagnosis but may even occur decades later [7]. Recurrent or metastatic disease is associated with aggressive behavior and a poor prognosis, as nearly 70% of patients developing recurrence will eventually succumb to their disease [8].

The optimal management of the relapsed disease is controversial due to the rarity of this entity and lack of randomized studies. Initial salvage therapy typically involves surgical debulking followed by cisplatin-based chemotherapy [7]. While impressive tumor responses may initially be achieved with chemotherapy or hormone therapy, they are durable for less than half of patients [5]. Furthermore, the tendency for prodigious growth may preclude patients from surgical debulking, leading to persistent pain and discomfort. Radiation therapy is an attractive option for providing palliation and improving local control without the morbidity of additional surgery or chemotherapy [9]. Unfortunately, the efficacy of radiation in this clinical context is not well described in the literature. Here we describe the tumor and biochemical response of a patient treated with palliative radiation for multiply recurrent, rapidly growing intraperitoneal GCT refractory to repeated surgical debulking and several lines of systemic therapy.

## Case presentation

A 69-year-old woman with a history of ovarian GCT presented with increased serum levels of inhibin B concerning for disease recurrence. Eight months previously, she was diagnosed with International Federation of Gynecology and Obstetrics (FIGO) stage IC GCT, which was initially managed with hysterectomy and bilateral salpingo-oopherectomy. Adjuvant chemotherapy was recommended but she ultimately declined. At the time of biochemical recurrence, restaging computed tomography (CT) of the chest, abdomen, and pelvis with IV contrast demonstrated multiple intraperitoneal nodules. Given her favorable performance status, exploratory laparotomy with cytoreduction was felt to be appropriate. At the time of surgery, multiple macroscopic tumor deposits measuring less than 2cm were discovered in the right lower quadrant at the surface of her ascending colon and inferior liver, upstaging her to FIGO IIIC. This was followed by six cycles of carboplatin and paclitaxel. Unfortunately, restaging scans showed progressive intra-peritoneal disease. Surgical debulking was repeated at that time, but the patient was reluctant to pursue additional platinum-based chemotherapy after developing neuropathy. Subsequently, she was started on letrozole followed by tamoxifen alternated with megestrol acetate.

Two months later, restaging PET/CT demonstrated a 3.7-cm hypodense lesion of the posterior segment of the right lobe of her liver with a standardized uptake value (SUV) of 5.9. Additionally, multiple sub-centimeter nodules were noted along the abdominal wall. She received three cycles of bevacizumab but further growth of the liver mass was noted on restaging scans three months later, although inhibin B was stable at 309pg/mL. The patient was referred to a surgical oncologist and underwent right hepatectomy and cholecystectomy. Final pathology of the liver mass demonstrated metastatic GCT of the ovary, upstaging her to FIGO stage IVB. Postoperatively, serum inhibin B levels were elevated at 529pg/mL.

**Figure 1:**
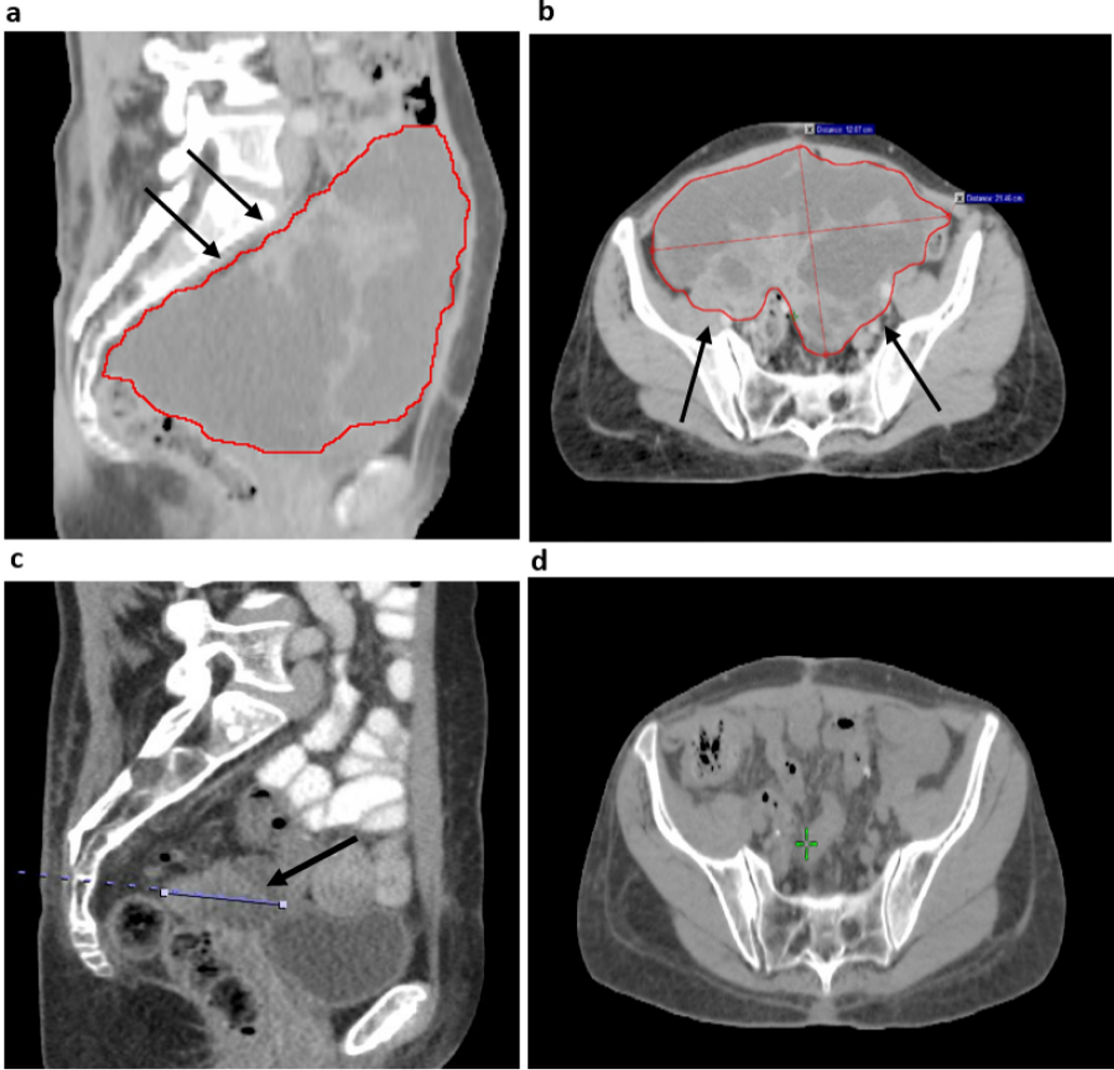
Recurrent intraperitoneal GCT before and after irradiation CT abdomen and pelvis demonstrating a large pelvic mass, outlined in red, in the (a) sagittal and (b) axial plane prior to starting radiation; images of the pelvis obtained three months after completing radiation therapy are depicted in (c) the sagittal view, where the maximum diameter is demarcated in blue, and (d) the axial view where the mass is no longer visible at this position. GCT: Granulosa cell tumor

Restaging scans performed one month later demonstrated progression of multiple intraperitoneal nodules. The patient received three cycles of gemcitabine. Shortly after completing systemic therapy, she was admitted for urgent management of progressively worsening early satiety, postprandial epigastric pain, nausea, vomiting, pelvic fullness, and severe constipation. On exam, a large suprapubic mass was palpable at the midline. At that time, serum inhibin B levels were 1,014pg/mL, nearly doubling over the course of two months. Abdominal CT demonstrated a rapidly progressing large, loculated, cystic pelvic mass measuring 21.5 x 12.1 x 13.3cm (Figures 1a, 1b), and another large left upper quadrant (LUQ) mass measuring 9.0 x 5.8 x 6.0cm. Given the severity of her symptoms and disease progression on multiple lines of systemic therapy, the patient was referred for consideration of palliative radiotherapy.

Based on institutional reports of effective palliation with radiotherapy for recurrent GCT [9,10], the patient was consented for palliative radiation to the two largest metastatic lesions described above. The larger 21cm pelvic mass was treated to a total dose of 46Gy at 2Gy per fraction for 23 fractions utilizing an intensity-modulated radiotherapy (IMRT) technique. The smaller LUQ mass was treated to a total dose of 40Gy at 3Gy per fraction for 15 fractions utilizing a volumetric modulated arc therapy (VMAT) technique. Radiation was well tolerated apart from grade 2 nausea, which was managed supportively. The patient reported a significant reduction of abdominal fullness over the course of treatment with improvement in appetite and PO intake. Inhibin B measured six weeks' post-treatment decreased from 1,014 pg/mL to 645pg/mL. The patient subsequently started topotecan.

**Figure 2:**
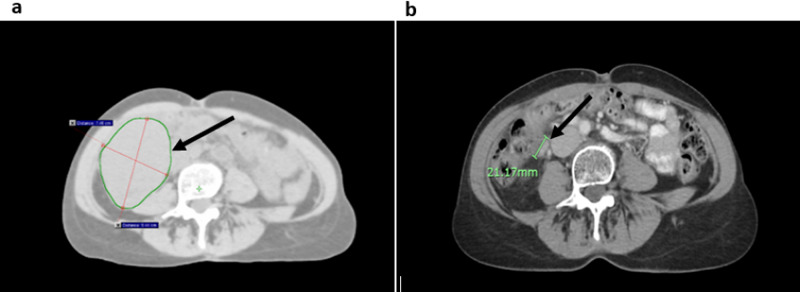
Recurrent right upper quadrant GCT before and after radiation therapy Axial CT abdomen and pelvis obtained (a) prior to radiation, demonstrating a large right upper quadrant mass outlined in green, and (b) three months after completing radiation, where the maximum tumor diameter is demarcated in green. GCT: Granulosa cell tumor

At three-month follow-up, inhibin B increased to 888pg/mL, and restaging CT was performed to assess treatment response. Based on imaging, the treated pelvic mass decreased significantly in size to 6.6 x 5.4 x 4.1cm (Figures 1c, 1d), achieving a 96% reduction in tumor volume. The treated LUQ mass also showed a substantial reduction in size measuring 3.9 x 3.0 x 2.9cm corresponding to a 92% reduction in tumor volume. However, an untreated nodule in the right upper quadrant (RUQ), adjacent to the inferior pole of the right kidney, had increased in size from 3.4 x 3.2 x 3.2cm at the time of imaging prior to starting radiation, to 9.4 x 7.4 x 8.7cm on restaging scans (Figure 2). At this time, there were no other radiographically apparent lesions. Topotecan was discontinued and the patient was consented for another course of palliative radiotherapy to the RUQ mass. In order to meet dose constraints to organs at risk, this was delivered to a dose of 36 Gy in 15 fractions utilizing a 3D-conformal arc technique.

**Figure 3:**
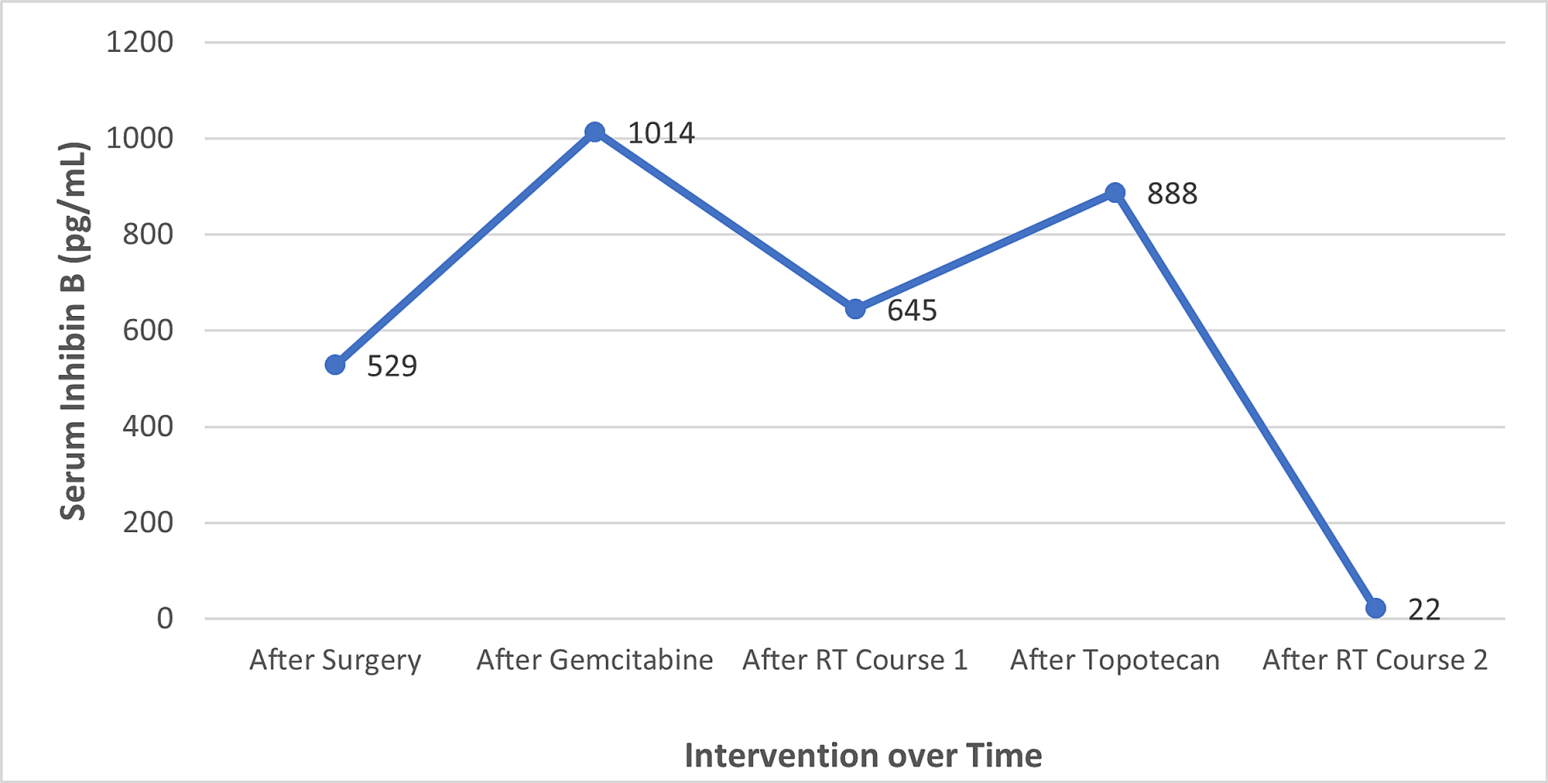
Concentration of serum inhibin B after each therapeutic intervention Line graph demonstrating serum inhibin B levels (blue line) following each intervention listed in chronological order over the course of one year.

At three-month follow-up, the patient reported significant improvement in the frequency and severity of her abdominal complaints. Restaging CT demonstrated a 92% volume reduction for the recently treated RUQ mass (Figure 2b). The pelvic and LUQ masses treated six months previously continued to respond to therapy, with both measuring less than 3cm in maximum diameter. There were no new metastatic lesions observed in the abdomen or pelvis. Serum inhibin B levels decreased to 22pg/mL, achieving a 98% reduction in response to palliative radiotherapy with no interim systemic therapy. Serum inhibin B levels after each intervention described above are chronologically depicted in Figure 3.

## Discussion

While early-stage GCT exhibits indolent behavior, recurrent GCT tends to be aggressive and consensus for optimal management has yet to be defined. Initial management for recurrent or metastatic GCT typically involves surgical debulking followed by adjuvant chemotherapy; however, the majority of these patients will progress and ultimately die from their disease [5]. Here we describe a patient presenting with rapidly progressive intraperitoneal recurrence after failing repeated surgical debulking and multiple lines of systemic therapy. The patient was treated with palliative radiotherapy, achieving significant reductions in tumor volume, decreased serum biomarker levels, and prompt symptomatic relief. Tumor progression did occur outside of the initially irradiated field, but this area was subsequently treated with a separate course of palliative radiotherapy to which it responded equally well. After completing radiation, treated tumor volumes decreased by more than 90% and were well controlled on subsequent imaging up to one year later. This is in accordance with the response described in a case report by Choan et al., where an ~90% reduction in tumor volume was achieved for three patients treated with palliative radiotherapy for recurrent GCT, with doses ranging from 30Gy in 20 fractions, to 55Gy in 30 fractions. The authors reported a durable response within the irradiated field at last follow-up, ranging from five to 21 months [10].

In addition to tumor volume, a concomitant reduction in serum inhibin B was observed in response to radiotherapy. Serum inhibin B concentration reflects tumor burden and often serves as a useful biomarker for disease recurrence and therapeutic response in patients with GCT. This was demonstrated in an investigation by Mom et al. where serum levels of inhibin A and B significantly declined in response to initial surgical intervention and were predictably elevated in the setting of disease recurrence [4]. Our case demonstrates a striking tumor biomarker response to palliative radiation for recurrent GCT. For our patient, serum inhibin B levels increased with disease progression through each course of systemic therapy, and in contrast, declined by 97% following palliative radiation to all sites of visible disease with no interim systemic therapy.

To our knowledge, this report is the first to describe serum tumor marker response, and only the second to describe the radiologic response of recurrent intraperitoneal GCT treated with palliative radiation therapy. Our case demonstrates that palliative radiation has the potential to offer rapid tumor volume reduction and excellent local control for recurrent GCT as demonstrated by radiographic studies and serum biomarker levels. Additionally, our patient experienced rapid symptomatic relief and improved quality of life in response to treatment. As mentioned by Choen et al., there are larger retrospective series demonstrating good clinical responses to radiotherapy; however, radiographic and biomarker responses were not reported [9-11]. Larger studies are needed to confirm the radiosensitivity of recurrent GCT and identify patient subsets that derive the most benefit from radiotherapy. As our patient had successfully resected oligometastatic disease and targetable intraperitoneal disease, the same degree of biochemical response may not be observed for patients with a greater tumor burden. Further, as disease progression outside of the irradiated field is common, optimizing systemic therapy in the recurrent setting remains crucial.

## Conclusions

Recurrent or metastatic GCT is associated with aggressive growth and a poor prognosis. The optimal management of the relapsed disease is controversial. Initial salvage therapy typically involves surgical debulking followed by cisplatin-based chemotherapy; however, less than half of patients achieve a durable response with this regimen. Here we present a case of symptomatic, multiply recurrent GCT refractory to repeated surgical debulking, and several lines of systemic therapy. The patient was treated with palliative radiotherapy, achieving significant reductions in tumor volume, decreased serum biomarker levels, and improved quality of life at one-year follow-up.
